# Water-Pipe Smoking and Albuminuria: New Dog with Old Tricks

**DOI:** 10.1371/journal.pone.0085652

**Published:** 2014-01-17

**Authors:** Iqra Ishtiaque, Kashif Shafique, Zia Ul-Haq, Abdul Rauf Shaikh, Naveed Ali Khan, Abdul Rauf Memon, Saira Saeed Mirza, Afra Ishtiaque

**Affiliations:** 1 University Medical and Dental College, Faisalabad, Pakistan; 2 School of Public Health, Dow University of Health Sciences, Karachi, Pakistan; 3 Institute of Health and Wellbeing, Public Health, University of Glasgow, Glasgow, United Kingdom; 4 Institute of Public Health and Social Sciences, Khyber Medical University, Peshawar, KPK, Pakistan; 5 Department of Community Medicine, Dow University of Health Sciences, Karachi, Pakistan; 6 Department of Surgery, Dow University of Health Sciences, Karachi, Pakistan; 7 Department of Medicine, Dow University of Health Sciences, Karachi, Pakistan; 8 Department of Epidemiology, University of Rotterdam, Rotterdam, The Netherlands; 9 King Edward Medical University, Lahore, Pakistan; The National Institute for Health Innovation, New Zealand

## Abstract

Water-pipe (WP) smoking is on rise worldwide for the past few years, particularly among younger individuals. Growing evidence indicates that WP smoking is as harmful as cigarette smoking. To date, most of the research has focused on acute health effects of WP smoking, and evidence remains limited when it comes to chronic health effects in relation to long-term WP smoking. Therefore, the aim of this study was to examine the association between WP smoking and albuminuria in apparently healthy individuals. This analysis was conducted on data of a population-based cross-sectional study—the Urban Rural Chronic Diseases Study (URCDS). The study sample was recruited from three sites in Pakistan. Trained nurses carried out individual interviews and obtained the information on demographics, lifestyle factors, and past and current medical history. Measurements of complete blood count, lipid profile, fasting glucose level, and 24-hour albuminuria were also made by using blood and urine samples. Albumin excretion was classified into three categories using standard cut-offs: normal excretion, high-normal excretion and microalbuminuria. Multiple logistic regression models were used to examine the relationship between WP smoking and albuminuria. The final analysis included data from 1,626 health individuals, of which 829 (51.0%) were males and 797 (49.0%) females. Of 1,626 individuals, 267 (16.4%) were current WP smokers and 1,359 (83.6%) were non-WP smokers. WP smoking was significantly associated with high-normal albuminuria (OR  =  2.33, 95% CI 1.68-3.22, p-value <0.001) and microalbuminuria (OR  =  1.75, 95% CI 1.18-2.58, p-value 0.005) after adjustment for age, sex, BMI, social class, hypertension, and diabetes mellitus. WP smoking was significantly associated with high-normal albuminuria and microalbuminuria when analysis was stratified on hypertension and diabetes mellitus categories. WP smoking has a strong association with albuminuria in apparently healthy individuals. More research is warranted to evaluate the temporality of this association between WP smoking and albuminuria.

## Introduction

Water-pipe (WP) also named as shisha, narghile, hookah and arghile is an ancient device which has had a re-birth in recent years [1], [2]. A perforated aluminium foil is used in a typical WP which splits the burning charcoal from tobacco. This charcoal and tobacco is connected to a water bowl through a pipe. The air is drawn through the mouthpiece of a hose when a smoker inhale it and this results in burning of both charcoal and tobacco, while the smoke bubbles through the water in this inhaling process [1], [2]. There is a misconception that the course of smoke over the water makes it “filtered” and its potential harmful effects on human health are reduced if not completely removed by this so-called “filtering” process [3], [4]. There are many similarities between traditional and modern WPs in terms of its configuration as well as the contents used in these devices. Perhaps the only difference between the old and modern WPs is that the charcoal and tobacco is more refined in the later while they were in raw form in the old WPs.

WP smoking trend has dramatically increased in recent years especially among younger individuals [2], [5]. Some variations in prevalence of WP smoking are also reported in different regions of the world such as: Arab Americans (12%-15%), Arabic Gulf region (9%-16%), Estonia (21%), and Lebanon (25%) [2], [5]. The highest prevalence was reported among University students of Syria (25.5%) [6] and it also remained fairly high (24.5%) among younger individuals (18-24 years of age) in the United States (US) [7]. Pakistan has also seen a rise in WP smoking in last few years with approximately 33% of student being current smokers and nearly half of the University students have tried it once. [8].

In recent years, harmful effects of WP smoking have been reported with particular attention to respiratory and cardiovascular effects [9], although the evidence is limited to acute harmful properties of WP smoking on cardio-respiratory health [9], [10]. A recent population-based study suggested that WP smokers had double risk of having metabolic syndrome compared to non-users with more pronounced effects among women [11]. The harmful effects of WP smoking are mainly attributed to toxicants in the smoke which are similar toxicants as in the cigarettes [1], [12]. Interestingly, some even suggested that the CO content in WP smoke is three to ten times higher than in cigarette smoke [1], [13], [14]. Furthermore, WP smokers showed significantly higher blood nicotine levels than cigarette smokers [15].

Urinary albumin excretion is a very sensitive marker of renal injury and its role has also been identified in risk of cardiovascular diseases among healthy as well as diabetic individuals [16], [17]. Cigarette smoking has been previously linked with albuminuria and abnormal renal function in humans [18]. Moreover, smoking less than and greater than 20 cigarettes per day shown increased risk of microalbuminuria (Relative risk 1.92, 95% CI 1.54-2.39) and (Relative risk 2.15, 95% CI 1.52-3.03) respectively, among healthy as well as diabetic individuals [19], [20].

Given that growing evidence now indicates that WP smoking is as harmful as cigarette smoking, its impact on other aspects of health need to be investigated. To date, most of the research has focused on acute health effects of WP smoking, and the scientific evidence on chronic conditions (cardiovascular and renal) and its association with long-term WP smoking remains scarce. If the toxicants in WP smoke are fairly similar to those of cigarette smoke, there may be a relationship between WP smoking and abnormal renal functions as cigarette smoking has well known adverse effects on kidneys. Therefore, the aim of this analysis was to determine the association between WP smoking and albuminuria in seemingly healthy individuals.

## Methods

### Cohort selection and study participants

This study was conducted using the data of the Urban Rural Chronic Diseases Study (URCDS) - a cross-sectional study conducted in urban and rural settings of Pakistan to examine the development and prognosis of chronic diseases. The details of this study design and characteristics of the study sample have been explained elsewhere [11]. In brief, the study sample was drawn from the northeast of Punjab province in Faisalabad city (an urban centre) and a village (a rural centre) nearly 35 kilometres away from the centre of the city. Faisalabad is third biggest city of Pakistan with a population of approximately six million when this cross-sectional survey was conducted. Three hospitals (two urban and a rural) were involved in data collection process i.e. Afra General Hospital, Haider Medicare Hospital and New Lahore Hospital.

To increase the participation rate of community in this baseline survey, potential participants were contacted using a variety of different approaches. Participants were requested to participate in this study by personalised letters using the regional voter list as a sampling frame which have individuals older than 18 years but we only sampled from those who were 30 years or above at the time of sampling. Simple random sampling was carried out through Stata software using voter list as sampling frame. Furthermore, announcements in community gatherings, study information dissemination through local key informants, political leaders and religious heads. Healthy adults of age 30 to 75 years living in the vicinity of above mentioned hospitals were eligible to participate in the study. Three clinics were especially set-up in hospitals and all participants who visited during the period of 1^st^ January 2006 to 31^st^ June 2009 were included in this study. Both males and females, employed or un-employed as well as retired from their jobs were eligible to be included in the study. Individuals accompanying patients for their routine health check-ups were also approached and included in the study, if consented.

All eligible individuals were interviewed by a trained staff nurse, followed by a physical examination by a medical specialist at specially set-up clinic. The interview included demographic detail (age, sex, occupation, income, area of residence), lifestyle habits (including cigarette smoking, water-pipe smoking and physical activity) and general health questions. Social class was used as a measure of socio-economic status and every individual was classified to a category based on their occupation. The classification included “Upper class”- including professional individuals holding a management level post in either private or government setup, “middleclass” was assigned to non-manual employees who were skilled office workers and “lower-class” was labelled to manual labour (unskilled workers or farmers). Women were either classified the social class of their husbands or parents depending on their marital status. Following the initial interview, physical examination for measurements of weight, height, waist circumference and blood pressure were taken. Blood samples were obtained by trained nurses for the measurements of lipid profile and glucose level. A subgroup of individuals was also investigated for C-reactive protein and albuminuria. Trained nurses provided oral as well as written information to participants about the method to collect 24-hour urine samples. Participants were also requested to delay the urine collection if they have fever, urinary tract infection or menstruation. They were also informed cease the heavy exercise at least for 24 hours of urine collection. Participants were requested to keep the urine cold (at 4°C) before the visit to clinic but not more than two days. Measurements of urine volume and albumin were obtained in respective laboratories attached to hospitals.

### Albuminuria definitions

We used the standard definition to define microalbuminuria i.e. an albumin excretion of 30 to 300 mg/24 hours. As some evidence also suggests that the risk of cardiovascular and renal diseases may rise even at a lower cut-off point than what is considered as pathologic, we also created a group of individuals who had albumin excretion between 15 to 30 mg/24 hours and classified them as high-normal albuminuria as in previously reported studies.

### Ethics statement

This study was reviewed and approved by the Ethical Review Committee at the, Afra General Hospital, Faisalabad. This study was also carried out in accordance with the Declaration of Helsinki guidelines. The approvals were also obtained from all three hospitals – Afra General Hospital, Haider Medicare Hospital and New Lahore Hospital. Study participants provided written informed consent prior to their participation in the study.

### Sample size estimation

The sample size for this study was estimated to detect a 2% difference in prevalence of albuminuria (assuming 20% prevalence of albuminuria among WP smokers) between groups. The significance level was at 0.05 and power of the study at 80% using two sided comparisons. Both *X^2^* using the Yates’ continuity correction and Fisher Exact Test were used to compute the sample size. A total of 1535 individuals were required to be included in the study to conduct this survey with an allocation ratio of 1.

### Data analysis

We used STATA software version 11 (StataCorp, College Station, TX, USA) for data analysis. The study sample was categorized into two groups such as “WP smokers” and “non-WP smokers”. Current water-pipe smokers were defined as those who regularly smoked WP at least once in a week in the previous year. Non-WP smoker group included those who were never WP smokers as well as who quit smoking (ex-WP smokers). Those who quitted WP smoking at least twelve months before the beginning of this study were defined as Ex-smokers those who quitted less than a year’s time were considered as smokers. Age was categorised into 10 year age bands except the highest age group which was a 15 years age band due to smaller number of individuals in that group.

For the estimation of raised blood pressure and glucose level, new variables were created using the IDF specific criteria. Hypertension was defined as systolic blood pressure of at least 140 mm Hg, diastolic blood pressure of at least 90 mm Hg, or history of use of antihypertensive drugs. Diabetes mellitus was defined as a fasting plasma glucose level of >126 mg/dL, a random plasma glucose level of >180 mg/dL or greater, or the use of antidiabetic medication. Therefore, wherever hypertension or diabetes mellitus is mentioned in this manuscript, it reflects increased level or previously known condition, or both.

Independent sample t-test was used to compare continuous variables between two groups while chi-square test was used for comparison between categorical variables. We used multiple logistic regressions to examine the association between WP smoking and high-normal albuminuria as well as microalbuminuria. Separate multivariable logistic regression models were ran to explore the association between WP smoking and high albuminuria and microalbuminuria after adjusting for age, sex, BMI, social class, hypertension and diabetes mellitus. A stratified analysis was also carried out to estimate the effect of WP smoking on albuminuria based on hypertension and diabetes mellitus categories.

A total of 2700 individuals were included in this study however there were some information missing i.e. blood pressure data (n = 75), self-reported diabetes mellitus and blood glucose (n = 47), height and weight (n = 24), social class and triglycerides level(n = 79), Those who had missing information for any of these were excluded from the final analysis. Furthermore, those who were cigarette smokers (n = 128) or combine users of cigarette and WP (n = 443) were also excluded from the present analysis. The data on albumin excretion was also not available for 406 individuals, so they were also excluded from this analysis.

## Results

Data from 1,626 healthy individuals were used for final analysis which included 829 (51.0%) males and 797 (49.0%) females. Of 1,626 individuals, 267 (16.4%) were current WP smokers and 1,359 (83.6%) were non-WP smokers. Number of ex-WP smokers was fairly small (n = 13), we performed a sub-analysis by excluding these ex-WP smokers but overall association between WP and albuminuria remained unchanged so they were included in the non-WP smoking category. There was no significant difference in duration of WP smoking among three groups of albumin excretion (p-value 0.20), while the overall mean duration of WP smoking was 14.9±10.8 years. Out of the total 1,626, 1011 (62.3%) had albumin excretion within normal range, 340 (20.9%) had high-normal albumin excretion and 273 (16.8%) had microalbuminuria. No statistically significant differences of age at baseline examination, sex, BMI and social class were observed between three groups of albumin excretion. Furthermore diabetics, hypertensives and current WP smokers were significantly more likely to have microalbuminuria compared with non-diabetics, normotensives and non-WP smokers, respectively. Table 1, shows the demographic and lifestyle characteristics of study sample.

**Table 1:** Baseline characteristics of study participants by albumin excretion.

		Albumin excretion			P-value*
		Normal	High-normal	Microalbuminuria	
		<15 mg/L	15-30 mg/L	>30-300 mg/L	
		(n = 1011)	(n = 340)	(n = 274)	
		n (%)	n (%)	n (%)	
**Age at screening (continuous)**		47.97±11.9	53.33±13.0	57.18±12.2	<0.001
**Age at screening (years)**					
	30-39	295 (76.2)	61 (15.8)	31 (8.0)	<0.001
	40-49	307 (71.9)	75 (17.6)	45 (10.5)	
	50-59	196 (57.1)	80 (23.3)	67 (19.6)	
	60-75	213 (45.6)	124 (26.6)	130 (27.8)	
**Sex**					
	Male	569 (68.7)	159 (19.2)	100 (12.1)	<0.001
	Female	442 (55.4)	181 (22.8)	173 (21.8)	
**Social class**					
	Professional	129 (50.6)	63 (24.7)	63 (24.7)	<0.001
	Non-manual workers	384 (60.6)	128 (20.2)	122 (19.2)	
	Manual workers	498 (67.7)	149 (20.3)	88 (12.0)	
**Body mass index (continuous)**		25.6±3.9	26.6±3.9	28.0±4.6	<0.001
**Body mass index (kg/m^2^)**					
	Desirable < 23	258 (72.7)	62 (17.4)	35 (9.9)	<0.001
	Overweight >23-27.5	500 (66.5)	155 (20.6)	97 (12.9)	
	Obese > 27.5	253 (48.9)	123 (23.8)	141 (27.3)	
**Blood pressure**					
	Normotensive	855 (69.6)	236 (19.2)	137 (11.2)	<0.001
	Hypertensive	156 (39.4)	104 (26.3)	136 (34.3)	
**Diabetes mellitus**					
	No	886 (66.7)	266 (20.0)	176 (13.3)	<0.001
	Yes	125 (42.2)	74 (25.0)	97 (32.8)	
**Waterpipe smoking**					
	Non smokers	891 (65.7)	25 (18.9)	209 (15.4)	<0.001
	Current smokers	120 (44.9)	83 (31.1)	64 (24.0)	

P-value was calculated using the chi-squared test.

### Water-pipe smoking and high-normal albuminuria

The frequency of high-normal albuminuria was significantly higher among WP smokers compared with non-WP smokers. Univariable analysis showed that WP smokers were 2.4 times higher risk to have high-normal albuminuria compared with non-WP smokers (OR  =  2.40, 95% CI .75-3.28, p-value <0.001). Furthermore on univariable analysis, individuals who were, older (p-value <0.001), females (p-value 0.002), obese (p-value 0.001), had diabetes mellitus (p-value 0.001) and hypertension (p-value 0.002) were significantly more likely to have high-normal albuminuria compared with youngers, males, desirable weight individuals, non-diabetics and normotensives respectively (table 2). Conversely, manual workers were 30% less likely to have high-normal albuminuria compared with professionals (OR  =  0.61, 95% CI 0.43-0.87, p-value 0.001). On multivariable analysis, WP smoking was significantly associated with high-normal albuminuria (OR  =  2.33, 95% CI 1.68-3.22, p-value <0.001) after adjustment for age, sex, BMI, social class, hypertension and diabetes mellitus (table 2).

**Table 2:** Relationship between water-pipe smoking and high-normal albumin excretion.

		Univariable analysis			Multivariable analysis$		
		Odds Ratio (95% CI)		p value	Odds Ratio (95% CI)		p value
**Waterpipe smoking**							
	Non smokers		1			1	
	Current smokers	2.40	(1.75, 3.28)	<0.001	2.33	(1.68, 3.22)	<0.001
**Age at screening (years)**							
	30-39		1			1	
	40-49	1.18	(0.81, 1.72)	0.38	1.09	(0.75, 1.60)	0.65
	50-59	1.97	(1.35, 2.88)	<0.001	1.66	(1.12, 2.47)	0.01
	60-75	2.82	(1.98, 4.01)	<0.001	2.08	(1.40, 3.09)	<0.001
**Sex**							
	Male		1			1	
	Female	1.47	(1.45, 1.88)	0.002	1.53	(1.18, 1.98)	<0.001
**Social class**							
	Professional		1			1	
	Non-manual workers	0.68	(0.48, 0.98)	0.04	0.96	(0.61, 1.50)	0.84
	Manual workers	0.61	(0.43, 0.87)	0.006	1.00	(0.60, 1.65)	0.23
**Body mass index (kg/m^2^)**							
	Desirable < 23		1			1	
	Overweight >23-27.5	1.29	(0.93, 1.80)	0.13	0.90	(0.64, 1.29)	0.57
	Obese > 27.5	2.02	(1.42, 2.87)	<0.001	1.28	(0.82, 2.02)	0.28
Blood pressure *							
	Normotensive		1			1	
	Hypertensive	2.42	(1.81, 3.21)	<0.001	1.65	(1.19, 2.28)	0.002
Diabetes mellitus *							
	No		1			1	
	Yes	1.97	(1.43, 2.71)	<0.001	1.19	(0.83, 1.71)	0.34

$All estimates were adjusted for age at screening, sex, BMI, social class blood pressure and diabetes mellitus. * indicate increased level of specific measure or a previously diagnosed condition.

### Water-pipe smoking and microalbuminuria

The frequency of microalbuminuria was significantly higher among WP smokers compared to non-WP smokers (p-value 0.001). Univariable analysis showed that WP smokers were 2.3 times more likely to have high microalbuminuria compared with non-WP smokers (OR  =  2.27, 95% CI 1.62-3.19, p-value <0.001). Furthermore on univariable analysis, older individuals (p-value <0.001), females (p-value 0.002), obese individuals (p-value 0.001), individuals with diabetes mellitus (p-value 0.001) and individuals with hypertension (p-value 0.002) were significantly more likely to have high microalbuminuria compared to younger, male, desirable weight, non-diabetics and normotensive individuals respectively (table 3). Conversely, manual workers were 64% less likely to have high-normal albuminuria compared with professionals (OR  =  0.36, 95% CI 0.25-0.53, p-value 0.001). On multivariable analysis, WP smoking was significantly associated with high microalbuminuria (OR  =  1.75, 95% CI 1.18-2.58, p-value 0.005) after adjustment for age, sex, BMI, social class, hypertension and diabetes mellitus (table 3).

**Table 3:** Relationship between water-pipe smoking and microalbuminuria.

		Univariable analysis			Multivariable analysis$		
		Odds Ratio (95% CI)		p value	Odds Ratio (95% CI)		p value
**Water-pipe smoking**							
	Non smokers		1			1	
	Current smokers	2.27	(1.62, 3.19)	<0.001	1.75	(1.18, 2.58)	0.005
**Age at screening (years)**							
	30-39		1			1	
	40-49	1.39	(0.86, 2.26)	0.18	1.07	(0.64, 1.77)	0.8
	50-59	3.25	(2.05, 5.17)	<0.001	1.86	(1.12, 3.08)	0.02
	60-75	5.81	(3.78, 8.92)	<0.001	2.64	(1.63, 4.30)	<0.001
**Sex**							
	Male		1			1	
	Female	2.23	(1.69, 2.93)	<0.001	2.22	(1.63, 3.01)	<0.001
**Social class**							
	Professional		1			1	
	Non-manual workers	0.65	(0.45, 0.94)	0.02	1.09	(0.69, 1.73)	0.36
	Manual workers	0.36	(0.25, 0.53)	<0.001	0.93	(0.54, 1.60)	0.78
**Body mass index (kg/m^2^)**							
	Desirable < 23		1			1	
	Overweight >23-27.5	1.43	(0.94, 2.16)	0.09	0.75	(0.48, 1.18)	0.22
	Obese > 27.5	4.11	(2.73, 6.18)	<0.001	1.75	(1.04, 2.95)	0.34
Blood pressure*							
	Normotensive		1			1	
	Hypertensive	5.44	(4.06, 7.29)	<0.001	2.91	(2.07, 4.09)	<0.001
Diabetes mellitus*							
	No		1			1	
	Yes	3.91	(2.86, 5.33)	<0.001	1.63	(1.22, 2.35)	0.01

$All estimates were adjusted for age at screening, sex, BMI, social class blood pressure and diabetes mellitus. * indicate increased level of specific measure or a previously diagnosed condition.

### Water-pipe smoking and albuminuria in relation to hypertension and diabetes mellitus

**Figure 1:**
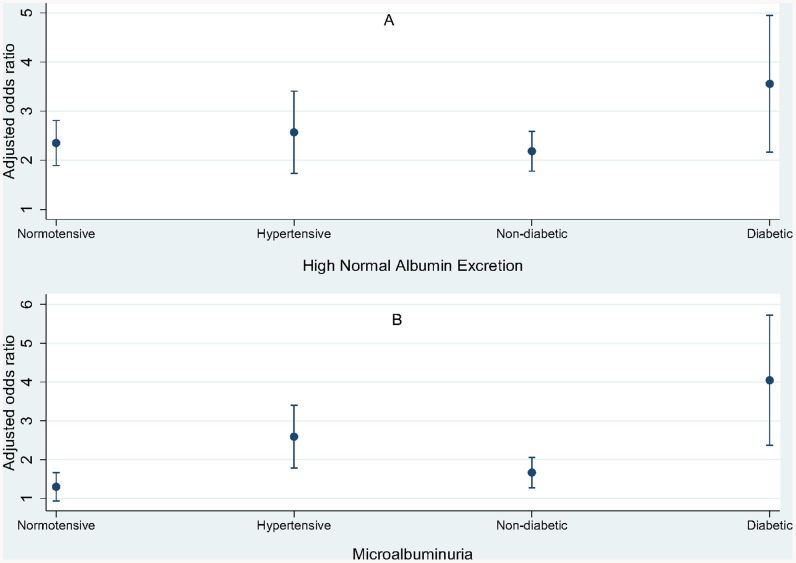
Association between and water-pipe smoking and albuminuria based on hypertension and diabetes mellitus categories. All estimates were adjusted for age, sex, BMI and social class.

A stratified analysis based on hypertension and diabetes mellitus status of individuals was also carried out to understand the relationship between WP smoking and albuminuria. WP smokers were two times more likely to have high-normal albuminuria compared with non-WP smokers regardless of hypertension status. However, WP smokers who had diabetes mellitus were 3.5 times more likely to have high-normal albuminuria compared to non-WP smokers; a slightly higher odds ratio compared to non-diabetics (figure 1A). The effect of WP smoking on microalbuminuria among diabetic and hypertensive individuals was higher when these effects were compared to non-diabetic and normotensive individuals (figure 1B).

## Discussion

This is the first population-based study providing some insights in to the effect of WP smoking on renal function of apparently healthy population. This study indicates a harmful relationship between WP smoking and albuminuria. The observed positive association between WP smoking and albuminuria was independent of age at baseline, sex, BMI, social class, hypertension and diabetes mellitus. The association between WP smoking and albuminuria changed a little when analysis was stratified on presence or absence of hypertension and diabetes mellitus.

Many previous studies have investigated the adverse influence of cigarette smoking on renal function but there is no previous evidence of detrimental effects of WP smoking on renal function. It is well established that the urinary albumin level is a sensitive marker of glomerular injury [19] and the fact that the cigarette smoking is associated with albuminuria indicates a direct or indirect relationship of cigarette smoking in initiation and progression of kidney damage [21]. The finding of this study that WP smoking is related to declining renal function is biologically plausible. Given that the WP smoke contains similar toxicants as cigarettes, kidney damage may have been a result of WP smoking through similar mechanisms as suggested for cigarette smoking. Several mechanisms have been proposed through which cigarette smoking can lead to renal injury.

The increase in glomerular filtration rate (GFR) caused by cigarette smoking may have some role in initiation and development of renal injury and chronic renal diseases [22], [23]. The increase in GFR due to cigarette smoking may possibly be a response to nicotine content of smoke which leads to increased excretion of sodium, chloride and urine flow [24], [25]. This observation also indicates that the release of catecholamine from adrenal medulla may have been responsible for nicotine action on the kidney. Another possible mechanism by which nicotine can alter GFR is the altered cervical parasympathetic tone may cause increase in vasopressin release which ultimately leads to increase in GFR [26], [27]. There is also a possibility that the increase in GFR may be a response to increased blood pressure secondary to smoking nicotine products. Other renal functions such as altered proximal tubular function, increased excretion of N-acetyl-f3-glucoasmindase and impairment of organic cation transport are also a consequence of cigarette smoking, which are particularly related with the amount of tobacco smoked over time. [28].

The findings of this study could be a result of some biases inherent in epidemiological studies. There is a chance of some selection bias which could be due to exclusion of those individuals who used to smoke both cigarettes and WP. The main reason for excluding these individuals was the inability to understand the true effect of WP smoke on albuminuria because of simultaneous cigarette smoking. Moreover, significant differences were observed in age at baseline examination, sex, BMI, social class, hypertension and diabetes mellitus of three study arms i.e. normal albumin excretion, high-normal albumin excretion and microalbuminuria. Differential distribution of these factors may have confounded the observed association between WP smoking and albuminuria, however, multivariable adjustment did not alter the findings of this study. Furthermore, stratified analyses were also carried out based on all these baseline factors but the relationship between WP smoking and albuminuria remained consistent (data not shown).

Diabetes mellitus and hypertension are independently associated with albuminuria so the stratified analyses were also carried out based on these variables. The overall associations between WP smoking and high-normal albuminuria were similar both in hypertension and diabetes mellitus categories. However, there was some evidence of differential effect of WP smoking on microalbuminuria with more pronounced effect among hypertensive and diabetic individuals. This may be combined effect of WP smoking with these morbidities to cause renal damage; a further study on healthy population without any morbidity may explain the true effect of WP smoking on microalbuminuria. Another important concern can be the exposure to second-hand smoke due to WPs, which may have influenced the findings of this study because WPs are usually smoked in social meetings with a few people around. Some participants of this study classified as non-WP smokers in this study may actually have been exposed to second-hand smoke through polluted air and may have developed impairment in their renal profile. This misclassification is not likely to change the overall outcome of this study because, if some of the non-WP smoker were actually exposed to second-hand smoke, the harmful effect of WP in this study may have been attenuated and underestimated while the actual effect of WP may even be greater than the one reported here.

This was the first population-based study with large sample size, which assessed the relationship between WP smoking and albuminuria. Generally, the adverse health effects of WP smoking are not very well understood yet and most of the health conditions are unexamined in association with WP smoking. Furthermore, the quality of evidence is fairly low which has been suggested by a reviews [29]. There are some limitations of this study as well which need to be accounted when drawing any conclusions from present analysis. Firstly, the study sample was drawn from one region of Pakistan which mainly represents only one ethnic group (which is approximately 60% of national population) living in this country so the findings of the present study may not be generalizable to other regions and ethnicities living in Pakistan. Secondly, selection of study sample was based on different types of invitations to increase the participation rate which may have led to some selection bias. However, our previous analysis has showed that the distribution of many risk factors i.e. obesity, hypertension and diabetes mellitus, is comparable with published studies from this region [11] so any effect is unlikely due to differential distribution of background risk factors in the study sample. Lastly, in this part of the country, traditional WPs are commonly used and these were mainly smoked by the study participants. There can be some argument that the raw charcoal and raw tobacco may not be similar in terms of smoke toxicants produced by the modern fashionable WPs. However, some evidence suggests that flavouring of tobacco and charcoal did not cut down the potential adverse effects of WPs [30]–[32].

### Public health implications

WP smoking has markedly increased globally in recent years. Given the injurious effects of WP smoking on renal function observed in this study, WP smoking may significantly increase the burden of renal diseases in future, however, confirmation of a causal relationship between WP smoking and renal damage is required before initiating wider public health actions. Further prospective studies including younger individuals and using different varieties of tobacco in WPs may be useful in understanding the relationship of WP smoking in development of renal and other chronic diseases. Health education and awareness programs specific to target groups are the only interventions which are critical to eliminate the false beliefs of WP harmlessness. Although, the target groups for such interventions can be broad but particular attention to younger age individuals will be important to reduce the future burden of diseases associated with WP smoking.

## Conclusion

This study observed a significant independent positive relationship between WP smoking and albuminuria among middle and old aged individuals. Future research using prospective cohort and including younger age individuals would be useful to understand the mechanism and temporal association between WP smoking and albuminuria.

## References

[1] Eissenberg T, Shihadeh A (2009) Waterpipe tobacco and cigarette smoking: direct comparison of toxicant exposure. Am J Prev Med 37: 518–523 10.1016/j.amepre.2009.07.014 19944918 PMC2805076

[2] Maziak W (2011) The global epidemic of waterpipe smoking. Addict Behav 36: 1–5 10.1016/j.addbeh.2010.08.030 20888700 PMC4135081

[3] Maziak W, Eissenberg T, Rastam S, Hammal F, Asfar T, et al (2004) Beliefs and attitudes related to narghile (waterpipe) smoking among university students in Syria. Ann Epidemiol 14: 646–654 10.1016/j.annepidem.2003.11.003 15380795

[4] Roskin J, Aveyard P (2009) Canadian and English students' beliefs about waterpipe smoking: a qualitative study. BMC Public Health 9: 10 10.1186/1471-2458-9-10 19134220 PMC2628878

[5] Akl EA, Gunukula SK, Aleem S, Obeid R, Jaoude PA, et al (2011) The prevalence of waterpipe tobacco smoking among the general and specific populations: a systematic review. BMC Public Health 11: 244 10.1186/1471-2458-11-244 21504559 PMC3100253

[6] Maziak W, Hammal F, Rastam S, Asfar T, Eissenberg T, et al (2004) Characteristics of cigarette smoking and quitting among university students in Syria. Prev Med 39: 330–336 10.1016/j.ypmed.2004.01.024 15226042

[7] Smith JR, Edland SD, Novotny TE, Hofstetter CR, White MM, et al (2011) Increasing hookah use in California. Am J Public Health 101: 1876–1879 10.2105/AJPH.2011.300196 21852640 PMC3222344

[8] Jawaid A, Zafar AM, Rehman TU, Nazir MR, Ghafoor ZA, et al (2008) Knowledge, attitudes and practice of university students regarding waterpipe smoking in Pakistan. Int J Tuberc Lung Dis 12: 1077–1084. 18713508

[9] Shaikh RB, Vijayaraghavan N, Sulaiman AS, Kazi S, Mohammed Shafi MS (2008) The acute effects of Waterpipe smoking on the cardiovascular and respiratory systems. J Prev Med Hyg 49: 101–107. 19278135

[10] Khabour OF, Alzoubi KH, Bani-Ahmad M, Dodin A, Eissenberg T, et al (2012) Acute exposure to waterpipe tobacco smoke induces changes in the oxidative and inflammatory markers in mouse lung. Inhal Toxicol 24: 667–675 10.3109/08958378.2012.710918 22906173 PMC3752682

[11] Shafique K, Mirza SS, Mughal MK, Arain ZI, Khan NA, et al (2012) Water-pipe smoking and metabolic syndrome: a population-based study. PLoS One 7: e39734 10.1371/journal.pone.0039734 22848361 PMC3407230

[12] Neergaard J, Singh P, Job J, Montgomery S (2007) Waterpipe smoking and nicotine exposure: a review of the current evidence. Nicotine Tob Res 9: 987–994 10.1080/14622200701591591 17943617 PMC3276363

[13] Daher N, Saleh R, Jaroudi E, Sheheitli H, Badr T, et al (2010) Comparison of carcinogen, carbon monoxide, and ultrafine particle emissions from narghile waterpipe and cigarette smoking: Sidestream smoke measurements and assessment of second-hand smoke emission factors. Atmos Environ 44: 8–14 10.1016/j.atmosenv.2009.10.004 PMC2801144 20161525

[14] Jacob P III, Abu Raddaha AH, Dempsey D, Havel C, Peng M, et al (2011) Nicotine, carbon monoxide, and carcinogen exposure after a single use of a water pipe. Cancer Epidemiol Biomarkers Prev 20: 2345–2353 10.1158/1055-9965.EPI-11-0545 21908725 PMC3210932

[15] Shafagoj YA, Mohammed FI, Hadidi KA (2002) Hubble-bubble (water pipe) smoking: levels of nicotine and cotinine in plasma, saliva and urine. Int J Clin Pharmacol Ther 40: 249–255. 12078938 10.5414/cpp40249

[16] Svensson MK, Cederholm J, Eliasson B, Zethelius B, Gudbjornsdottir S (2013) Albuminuria and renal function as predictors of cardiovascular events and mortality in a general population of patients with type 2 diabetes: A nationwide observational study from the Swedish National Diabetes Register. Diab Vasc Dis Res 10: 520–529 10.1177/1479164113500798 24002670

[17] Doggen K, Nobels F, Scheen AJ, Van Crombrugge P, Van Casteren V, et al (2013) Cardiovascular risk factors and complications associated with albuminuria and impaired renal function in insulin-treated diabetes. J Diabetes Complications 27: 370–375 10.1016/j.jdiacomp.2013.02.008 23537603

[18] Orth SR, Ritz E (2002) The renal risks of smoking: an update. Curr Opin Nephrol Hypertens 11: 483–488. 12187311 10.1097/00041552-200209000-00002

[19] Orth SR (2002) Smoking and the kidney. J Am Soc Nephrol 13: 1663–1672. 12039997 10.1097/01.asn.0000018401.82863.fd

[20] Pinto-Sietsma SJ, Mulder J, Janssen WMT, Hillege HL, de Zeeuw D, et al (2000) Smoking is related to albuminuria and abnormal renal function in nondiabetic persons. Ann Intern Med 133: 585–591. 11033585 10.7326/0003-4819-133-8-200010170-00008

[21] Orth SR, Hallan SI (2008) Smoking: a risk factor for progression of chronic kidney disease and for cardiovascular morbidity and mortality in renal patients--absence of evidence or evidence of absence? Clin J Am Soc Nephrol 3: 226-236. Smoking is related to albuminuria and abnormal renal function in nondiabetic persons. Ann Intern Med 133: 585–59110.2215/CJN.03740907. 10.2215/CJN.03740907 18003763

[22] Hostetter TH (1995) Progression of renal disease and renal hypertrophy. Annu Rev Physiol 57: 263–278 10.1146/annurev.ph.57.030195.001403 7778868

[23] Neuringer JR, Brenner BM (1993) Hemodynamic theory of progressive renal disease: a 10-year update in brief review. Am J Kidney Dis 22: 98–104. 8322801 10.1016/s0272-6386(12)70174-9

[24] Cooper RG (2008) Renal function in male Sprague-Dawley rats concurrently exposed to long-term nicotine (3-{1-methyl-2-pyrrolidinyl}pyridine) and methylated spirits (methyl alcohol). Ren Fail 30: 107–114 10.1080/08860220701742179 18197551

[25] Tamaoki L, Oshiro-Monreal FM, Helou CM (2009) Effects of nicotine exposure on renal function of normal and hypercholesterolemic rats. Am J Nephrol 30: 377–382 10.1159/000235622 19684386

[26] Andersson K, Siegel R, Fuxe K, Eneroth P (1983) Intravenous injections of nicotine induce very rapid and discrete reductions of hypothalamic catecholamine levels associated with increases of ACTH, vasopressin and prolactin secretion. Acta Physiol Scand 118: 35–40 10.1111/j.1748-1716.1983.tb07237.x 6312746

[27] Lightman S, Langdon N, Todd K, Forsling M (1982) Naloxone increases the nicotine-stimulated rise of vasopressin secretion in man. Clin Endocrinol (Oxf) 16: 353–358. 7094361 10.1111/j.1365-2265.1982.tb00727.x

[28] Wong LT, Smyth DD, Sitar DS (1992) Interference with renal organic cation transport by (-)- and (+)-nicotine at concentrations documented in plasma of habitual tobacco smokers. J Pharmacol Exp Ther 261: 21–25. 1560368

[29] Warnakulasuriya S (2011) Waterpipe smoking, oral cancer and other oral health effects. Evid Based Dent 12: 44–45 10.1038/sj.ebd.6400790 21701545

[30] Al Ali R, Rastam S, Ibrahim I, Bazzi A, Fayad S, et al.. (2013) A comparative study of systemic carcinogen exposure in waterpipe smokers, cigarette smokers and non-smokers. Tob Control. doi: 10.1136/tobaccocontrol-2013-051206. 10.1136/tobaccocontrol-2013-051206 PMC4136964 23988862

[31] Cobb CO, Shihadeh A, Weaver MF, Eissenberg T (2011) Waterpipe tobacco smoking and cigarette smoking: a direct comparison of toxicant exposure and subjective effects. Nicotine Tob Res 13: 78–87 10.1093/ntr/ntq212 21127030 PMC3107609

[32] Daher N, Saleh R, Jaroudi E, Sheheitli H, Badr T, et al (2010) Comparison of carcinogen, carbon monoxide, and ultrafine particle emissions from narghile waterpipe and cigarette smoking: Sidestream smoke measurements and assessment of second-hand smoke emission factors. Atmos Environ (1994 ) 44: 8–14 10.1016/j.atmosenv.2009.10.004 20161525 PMC2801144

